# Correlation of Memory T Cell Responses against TRAP with Protection from Clinical Malaria, and CD4^+^ CD25^high^ T Cells with Susceptibility in Kenyans

**DOI:** 10.1371/journal.pone.0002027

**Published:** 2008-04-30

**Authors:** Stephen M. Todryk, Philip Bejon, Tabitha Mwangi, Magdalena Plebanski, Britta Urban, Kevin Marsh, Adrian V. S. Hill, Katie L. Flanagan

**Affiliations:** 1 Centre for Clinical Vaccinology and Tropical Medicine, Oxford University, Churchill Hospital, Oxford, United Kingdom; 2 Kenya Medical Research Institute (KEMRI)–Wellcome Trust Collaborative Research Programme, Centre for Geographic Medicine Research (Coast), Kilifi, Kenya; 3 The Wellcome Trust Centre for Human Genetics, Oxford, United Kingdom; 4 Department of Immunology, Monash University, Clayton, Victoria, Australia; 5 Medical Research Council (MRC) Laboratories, Fajara, The Gambia; 6 School of Applied Sciences, Northumbria University, Newcastle-upon-Tyne, United Kingdom; University of California Los Angeles, United States of America

## Abstract

**Background:**

Immunity to malaria develops naturally in endemic regions, but the protective immune mechanisms are poorly understood. Many vaccination strategies aim to induce T cells against diverse pre-erythrocytic antigens, but correlates of protection in the field have been limited. The objective of this study was to investigate cell-mediated immune correlates of protection in natural malaria. Memory T cells reactive against thrombospondin-related adhesive protein (TRAP) and circumsporozoite (CS) protein, major vaccine candidate antigens, were measured, as were frequencies of CD4^+^ CD25^high^ T cells, which may suppress immunity, and CD56^+^ NK cells and γδ T cells, which may be effectors or may modulate immunity.

**Methodology and Principal Findings:**

112 healthy volunteers living in rural Kenya were entered in the study. Memory T cells reactive against TRAP and CS were measured using a cultured IFNγ ELISPOT approach, whilst CD4^+^ CD25^high^ T cells, CD56^+^ NK cells, and γδ T cells were measured by flow cytometry. We found that T cell responses against TRAP were established early in life (<5 years) in contrast to CS, and cultured ELISPOT memory T cell responses did not correlate with *ex-vivo* IFNγ ELISPOT effector responses. Data was examined for associations with risk of clinical malaria for a period of 300 days. Multivariate logistic analysis incorporating age and CS response showed that cultured memory T cell responses against TRAP were associated with a significantly reduced incidence of malaria (p = 0.028). This was not seen for CS responses. Higher numbers of CD4^+^ CD25^high^ T cells, potentially regulatory T cells, were associated with a significantly increased risk of clinical malaria (p = 0.039).

**Conclusions:**

These data demonstrate a role for central memory T cells in natural malarial immunity and support current vaccination strategies aimed at inducing durable protective T cell responses against the TRAP antigen. They also suggest that CD4^+^ CD25^high^ T cells may negatively affect naturally acquired malarial immunity.

## Introduction

Natural immunity that develops against *P.falciparum* malaria in endemic regions, providing protection from infection and/or disease, has yet to be clearly characterised. Acquisition of immunity against parasitaemia or disease is complicated. The stages of the parasite life cycle express different antigens against which the immune system responds with various effector mechanisms. Studying natural immunity not only improves our understanding of the pathogen-host interaction but may also provide clues for the design of malaria vaccines in terms of target antigens and optimal types of immunity. The role of immune responses that are not adaptive in nature, such as NK cells, γδ T cells should also be considered, as well as naturally-occurring CD4^+^CD25^high^ regulatory T cells of unknown specificity. NK cells and γδ T cells may have direct effector functions or may modulate other immune responses [1], [2], whilst naturally-occurring CD4^+^CD25^high^ FOXP3^+^ regulatory T cells may be suppress immunity [3], [4], [5]. Different individuals will randomly possess varying levels of all these cells. T cells against the liver stage of the malaria life cycle are one way in which sterile immunity may be effected, by eliminating infected hepatocytes or disrupting parasite development. This is considered the major mechanism by which immunisation with irradiated sporozoites elicits sterile protection in humans [6], as well as mediating the protective effect of various vaccines in animal models [7]. A number of studies in the field have implicated T cell responses to the pre-erythrocytic/liver stage antigen circumsporozoite (CS) protein as being associated with protection against malaria [8], [9], [10]. Thrombospondin-related adhesive protein (TRAP) is another pre-erythrocytic antigen that is a target for T cells and is one of the very few antigens that has generated protective immunity as a subunit vaccine in humans, and the only one in which such protection can be clearly attributed to vaccine-induced T cell responses [11]. Field studies in Africa have identified CD4^+^ and CD8^+^ T cell responses to TRAP induced by natural infection [12], and other studies suggest that TRAP is under diversifying selective pressure [13] further supporting a possible role in immune protection.

IFNγ-secreting T cells are a major effector mechanism for elimination of infected hepatocytes [14], together with cytotoxic T cells [15]. Immediate effector T cell responses can be measured in an *ex-vivo* 18 hour IFNγ ELISPOT assay, and are thought to comprise mainly effector-memory T cells which circulate shortly after antigenic priming or recall [16]. Resting memory T cells require antigenic re-stimulation and we have utilised an assay to measure such memory T cells [17], known as the “cultured IFNγ ELISPOT”, which reflects a potential IFNγ-secreting T cell capacity within PBMC. We have shown this assay to be capable of measuring a population of T cells distinct from effector responses [18], which appear to correlate more closely with protection from malaria following TRAP vaccination [19].

In this study we aimed to assess cell-mediated immunity as it relates to immune protection against malarial disease. We have previously examined *ex-vivo* T cell responses to TRAP in a cohort of subjects in a malaria-endemic region of coastal Kenya [20]. The study showed no relationship between *ex-vivo* IFNγ ELISPOT response against TRAP peptides, measured before a transmission season, and reduced malaria incidence in the following year. We now wished to investigated whether there was a relationship between resting memory T responses, as measured by cultured IFNγ ELISPOT against TRAP and CS, and subsequent development of clinical malaria, using stored cells from the same Kenyan cohort. We assessed how responses against these antigens, measured in different assays, relate to one another and to subject age. We also investigated potential correlations of the level of CD4^+^ CD25^high^ T cells , NK cells, and γδ T cells with variable protection. We found that cultured responses to TRAP but not CS were significantly associated with reduced malaria, whilst increased numbers of CD4^+^ CD25^high^ T cells were associated with increased malaria.

## Methods

### Subjects, Study Site and Follow up

Healthy volunteers between 1 month and 81 years of age were recruited from the Kenyan coastal district of Ngerenya. Informed consent (Consent form Text S2) was obtained from all subjects or their parent/guardian prior to the donation of 5 mls of venous blood into heparin anti-coagulant. Blood was taken in the first two weeks of September 1998, outside of the peak transmission periods. Ngerenya district is an area of moderate malaria transmission with an entomological inoculation rate of 10 infectious bites per person per year [21]. Ethical approval for this study (Ethical approval Text S1) was obtained from the Kenyan Medical Research Institute National Ethics Committee, as described previously [20]. Active follow up was performed for up to one year, with each participant visited weekly. Data for the entire cohort was analysed for 300 days after the early September start point. Those reporting subjective fever and/or having a documented temperature >37.5°C had finger prick blood smears prepared at Kilifi District Hospital, where parasite densities were counted. Clinical malaria was defined as a fever and/or temperature >37.5°C together with >2500 parasites/µl for those >1 year or <15 years of age, or any parasitaemia with fever and/or temperature >37.5°C in those under 1 year of age or >15 years, as determined in a previous study of this population [22]. A description of the subject population giving breakdown of ages represented and malaria incidence during the study are shown in Table 1. Due to limitations on sample, all tests could not be carried out on all volunteer samples (Table 2).

**Table 1:** Descriptive data on age groups tested and malaria incidence.

*Age range*	*Cultured TRAP*	*Cultured CS*	*FACS*	*No malaria*	*Malaria episode*
0–5	22*	21	28	9	20
5–10	34	26	41	14	30
10–20	20	18	25	12	15
>20	17	14	16	11	7
Total (n)	94	80	112	46	72

* number of individuals in age group per test or according to malaria incidence

**Table 2:** Association between immune parameters and incidence of malaria.

Survival (cont. variable):		Univariate			Multivariate		
***Response***	***HR***	***95% CI***	***p***	***HR***	***95% CI***	***p***	***n***
Cultured TRAP	0.85	0.62–1.20	0.31	0.70	0.47–1.03	0.068	94
Cultured CS	1.07	0.76–1.51	0.69	1.33	0.88–2.032	0.176	80
CD4^+^CD25^high^	1.55	0.91–2.63	0.11	2.65	0.75–9.36	0.130	108
CD56^dim^	0.53	0.25–1.12	0.098	0.35	0.076–1.57	0.17	112
CD56^bright^	0.95	0.55–1.65	0.55	1.08	0.32–3.64	0.9	112
γδ T cells	1.30	0.42–4.06	0.65	2.5	0.55–11.34	0.24	64
**Logistic:**							
***Response***	***OR***	***95% CI***	***p***	***OR***	***95% CI***	***p***	***n***
Cultured TRAP	0.68	0.41–1.12	0.13	0.50	0.27–0.93	**0.028**	94
Cultured CS	1.13	0.67–1.89	0.64	1.65	0.83–3.28	0.16	80
CD4^+^CD25^high^	2.12	0.92–4.91	0.079	2.52	1.05–6.08	**0.039**	108
CD56^dim^	0.27	0.076–1.01	0.076	0.33	0.084–1.29	0.11	112
CD56^bright^	0.80	0.33–1.94	0.63	0.70	0.28–1.78	0.46	112
γδ T cells	1.39	0.21–9.43	0.74	2.03	0.24–17.03	0.512	64

Univariate and Multivariate analysis with both Cox survival and logistic models were used to examine the data. HR hazards ratio, OR odds ratio, CI confidence interval, **p<0.05 in bold**

### T cell responses

Peripheral blood mononuclear cells (PBMCs), separated from whole blood on a lymphoprep (Axis-Shield, UK) gradient, were washed and resuspended in RPMI 1640 medium (Sigma, UK) supplemented 1 in 100 with penicillin/streptomycin, L-glutamine (Invitrogen, UK) and 10% FCS (Biosera, UK). Cells were set up in 1 ml cultures, in 24-well plates, at 1×10^6^/ml with peptides at 5–10 µg/ml, as optimized and described previously [19]. On days 3 and 7, 0.5 ml of culture medium was removed and replaced with medium containing 100 U/ml IL-2 (Chiron), resulting in a final concentration of 50 U/ml. On day 9 the cells were washed 3 times in medium and resuspended in 1 ml, and rested over night, before proceeding to a IFNγ ELISPOT assay. IFNγ ELISPOT kits were purchased from Mabtech (Sweden) and manufacturer's instructions followed, with modifications as previously described [19]. Antigenic stimuli used *in vitro* consisted of a pool of 57 peptides spanning TRAP and 48 peptides comprising CS (20mers overlapping by 10aa). Medium-only and PHA controls were used in all assays. Results are expressed as spot-forming cells (SFCs)/10^6^ PBMCs (net antigen-stimulated spots less medium) on day 10 restimulation or *ex-vivo*.

### Intracellular cytokine staining (ICS) and Flow Cytometry

Following the culture period, cell samples were examined for antigen-stimulated cytokine production by ICS, as previously optimized for mixed CD4/CD8 T cell responses [23], and recapitulating the cultured ELISPOT assay. Cells on day 10 were stimulated with peptides, or were unstimulated, for 20 hours, the last 18 hours of which were in the presence of 0.1 µg/ml Brefeldin A. Cells were washed, stained with surface antibodies (CD3, CD4, CD8) for 20 minutes before further washing and addition of Perm/Fix for 20 mins. After washing in perm/wash anti-IFNγ antibody was added for 20 minutes, and the cells were washed in PBS and examined using a FACScalibur. 100,000 cells were acquired. To examine other cell types in *ex-vivo* (uncultured) PBMC, cells were washed and stained for 20 mins with combinations of the following antibodies: CD56, CD3, CD4, CD25, γδ T cell receptor. For FOXP3 expression intracellular staining (ebiosciences, USA) as above was preceded by CD4, CD25 and CD127 staining. Appropriate isotype controls (mouse IgG2a-FITC, IgG1-PE, IgG1-APC, rat IgG2a-PE-Cy5) were used in each experiment (Figure S2). 50,000 cells were acquired on the FACScalibur. Data was analysed using Cellquest and FlowJo programs. All antibodies, Brefeldin A and ICS kit were purchased from BD (Oxford, UK).

### Statistics

Multivariate analysis was carried out on data using both logistic and Cox survival models. Survival was additionally analysed using stratified Kaplan-Meier graphs and the log-rank test. Spearman's test was used for correlation. Stata 9 (StataCorp, Texas, USA) was used for all analysis. The cut-off value for statistical significance was p<0.05.

## Results

### Relating cultured IFNγ ELISPOT to age, and to ex-vivo IFNγ ELISPOT

**Figure 1:**
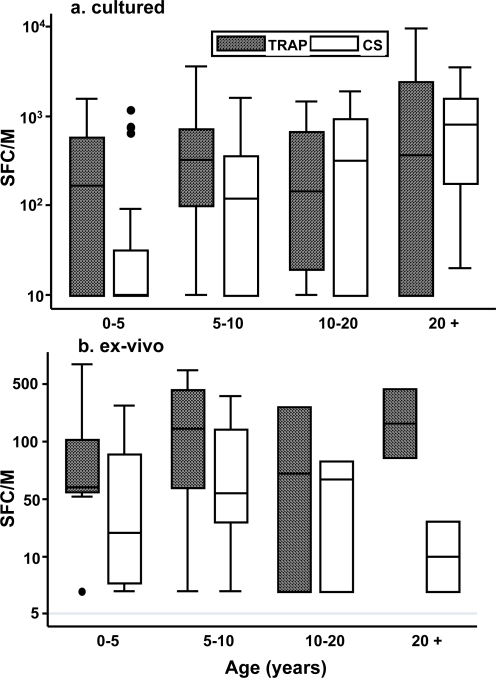
Relationship between ELISPOT responses and age. Cultured ELISPOT responses against (a) TRAP and (b) CS for individuals were stratified according to age into 4 groups: 0–5, 5–10, 10–20, and over 20. Median, 25^th^ and 75^th^ quartile, 5^th^ and 95^th^ quartile and outlying points are given by box and whisker plots.

**Figure 2:**
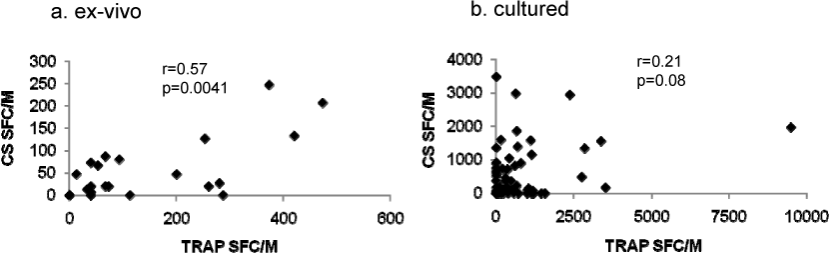
Correlations between ELISPOT responses. The relationship was examined between (a) TRAP and CS responses both ex-vivo, and (b) TRAP and CS responses both cultured.

The cultured responses of individuals were stratified according to age into 4 groups: 0–5, 5–10, 10–20, and over 20. TRAP cultured ELISPOT responses were already high (mean 352 spot-forming cells (SFC)/M PBMC) in 0–5 year old children, and did rise further (517 SFC/M) in 5–10 year olds, but not significantly. In contrast, CS responses were lower in young (0–5 yr) children (137 SFC/M), and rose significantly with age (10–20 yr; 541 SFC/M)) (Fig. 1a). For the limited number of *ex-vivo* responses carried out, no significant rise was seen for TRAP or CS (Fig. 1b). Dot plots for these responses are shown in supporting information (Figure S1). Those individuals for whom sufficient PBMC were available were tested for cultured and *ex-vivo* responses against TRAP (n = 23) or CS (n = 14), and no correlation between the responses was observed for either TRAP (p = 0.61) or CS (p = 0.76) (data not shown). However, the *ex-vivo* TRAP and CS responses did correlate significantly (n = 23) (r = 0.57, p = 0.0041)(Fig. 2c), and there was a non-significant trend towards a positive correlation between cultured TRAP and CS responses (n = 70, r = 0.21, p = 0.08) (Fig. 2d).

### Post Culture ICS and Surface Phenotyping

**Figure 3:**
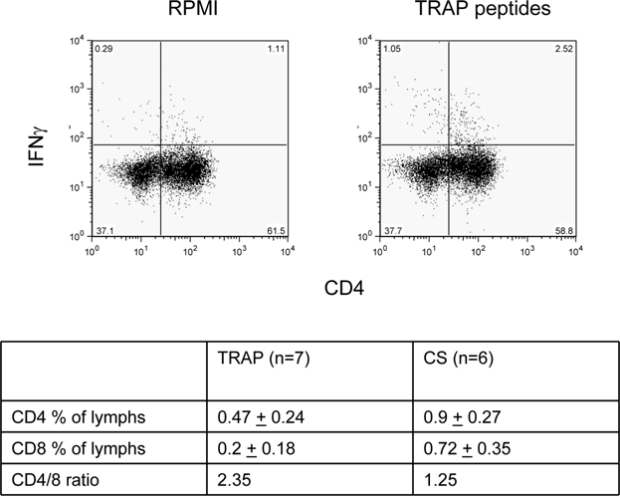
Intracellular IFNγ Staining following culture. ICS for IFNγ was carried out on cells by restimulation with peptides, following 10 days of culture, and co-staining with CD4 or CD8. The mean percentages (±standard deviation) of IFNγ^+^ cells possessing CD4 or CD8 (with medium controls subtracted) are shown together with the mean CD4:CD8 ratio.

In order to determine the phenotype of T cells responding to antigens in the cultured ELISPOT assay, ICS was carried out on day 10 of the culture process. Cultured cells were stimulated with peptides, or unstimulated, and then stained for surface CD4 and CD8 and intracellular IFNγ. When unstimulated responses (RPMI only) were compared to peptide-stimulated, a significant increase was seen for both TRAP (p = 0.03) and CS (p = 0.004). For TRAP-responding T cells, 0.47% of lymphocytes that were IFNγ^+^ were CD4^+^ whilst 0.2% were CD8^+^, i.e. a 2.4 fold excess of CD4^+^ over CD8^+^ (n = 7)(Fig. 3). For CS the fold excess of CD4^+^ over CD8^+^ was 1.3.

**Figure 4:**
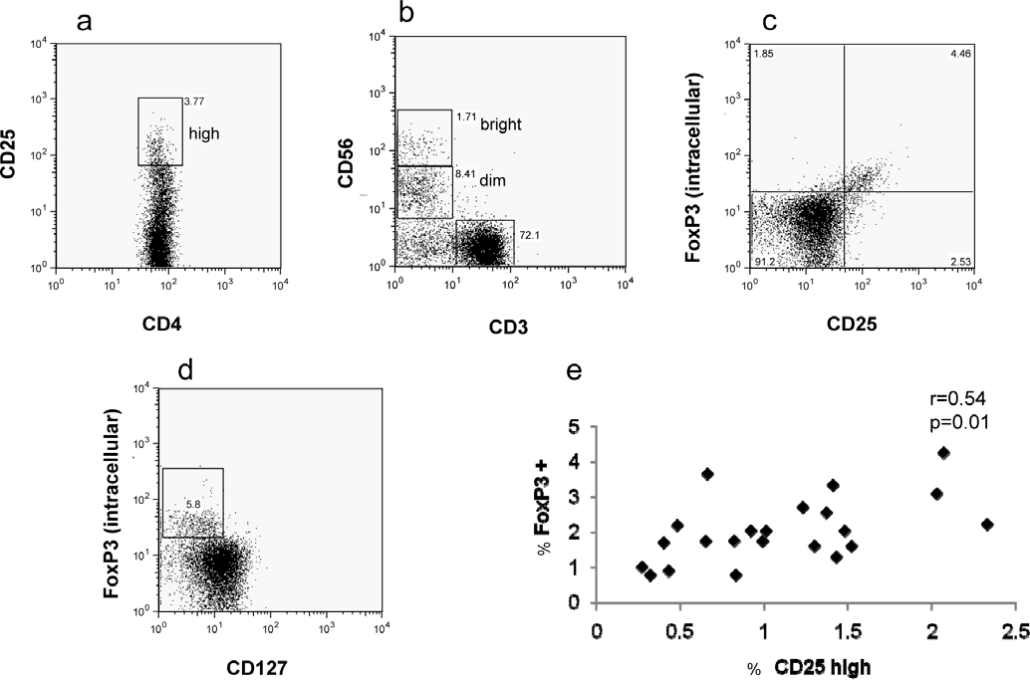
Characterisation of regulatory T cells and NK cells. Ex-vivo PBMC were stained for surface CD4, CD25 and CD127, and intracellularly for FoxP3. Typical dot plots show (a) the distribution of CD25 determining CD25^high^ status, (b) the distribution of CD56 on CD3 negative (NK) cells determining CD56^dim^ and CD56^bright^ status, (c) that the majority of FoxP3^+^ cells are CD25^high^, and (d) that the FoxP3^+^ cells are predominantly CD127^−^ [28]. (e) A correlation was demonstrated between CD25^high^ and FoxP3 positivity.

Unstimulated (*ex-vivo*) PBMC were examined for expression by lymphocytes of CD4/CD25, CD56 and γδ T cell receptor. Typical plots for CD4/CD25 and CD56 expression are shown (Fig. 4a,b), demonstrating CD4^+^ CD25^high^, and CD56^dim^/CD56^bright^ status. The CD4^+^CD25^high^ populations for a sub-group of the subjects were examined in more detail using intracellular staining for FOXP3 (Fig. 4c), where the majority of FOXP3^+^ T cells were CD25^high^, and for CD127 surface expression, where the majority were low/negative (Fig. 4d). Since a close correlation between CD25^high^ and FOXP3^+^ was seen (Fig. 4e), CD25^high^ was used for all further analysis.

### Associations with malaria incidence

The initial univariate analyses of the relationship between immune response and malaria (Table 2), showed a tendancy for cultured TRAP response (p = 0.13) and CD56^dim^ population (p = 0.076) to be associated with less malaria, and for CD4^+^ CD25^high^ cells with increased malaria (p = 0.079). A similar pattern was seen for logistic and Cox survival analyses. When a multivariate model was applied, adjusting for age and including both cultured CS and TRAP responses, the association between TRAP responses and protection became stronger (p = 0.068 for Cox survival, p = 0.028 for logistic regression), whilst multivariate analysis showed a significant association between increased CD4^+^ CD25^high^ population and increased malaria risk (p = 0.039).

**Figure 5:**
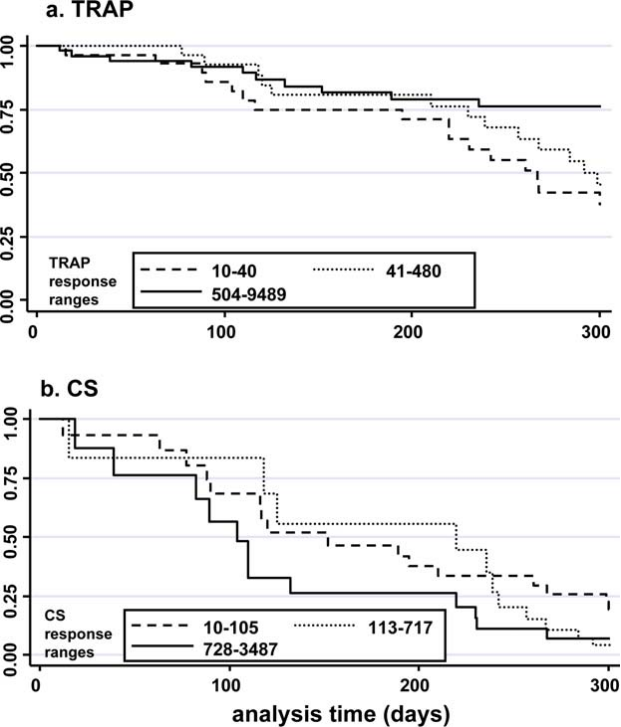
Kaplan-Meier malaria-free survival plots. Individuals were stratified into low, medium and high cultured ELISPOT responders (SFC/10^6^) to (a) TRAP and (b) CS and probability of remaining free of clinical malaria is plotted over the 300 day monitoring period. Co-variants incorporated into the analysis are age and antigen response to the other antigen.

The adjusted survival functions of level of cultured ELISPOT response to TRAP or CS is shown for individuals stratified into three tertiles according to response, as Kaplan-Meier graphs (Fig. 5). When analysed by tertile, a similar pattern was seen to the previous analysis, with a significant association with reduced malaria observed for cultured ELISPOT response to TRAP (Fig. 5a)(p = 0.046), in contrast to CS (Fig. 5b)(p = 0.20).

Multivariate analysis of the possession CD4^+^CD25^high^ T cells showed a significant association with increased malaria incidence (p = 0.039). Since CD4^+^CD25^high^ cells are known to have inhibitory effects on T cell responses, including proliferation, this population was compared to cultured ELISPOT responses by regression analysis. No negative or positive association between their frequency and cultured ELISPOT responses to TRAP (p = 0.9) or CS (p = 0.62) was observed (data not shown). No significant associations with survival were observed for the numbers of γδ T cells.

## Discussion

The search for immune responses and parameters that may contribute to natural immunity and influence the incidence of infection and/or disease is important, despite the difficulties in obtaining blood samples from carefully monitored individuals, particularly of young age, in regions where malaria is endemic.

Our initial study on this cohort of individuals measured *ex-vivo* T cell responses against selected TRAP peptides and showed no association between responses and protection [20]. Previous data suggests that *ex-vivo* and cultured ELISPOT assays measure distinct populations of responder T cells [18], [19], possibly due to differing priming and homeostatic mechanisms. Further, it appeared that cultured responses may associate better with protection from malaria both in malaria-naïve individuals following TRAP vaccination in the UK [19] and in studies of responses to the CS protein in The Gambia [9]. This study therefore aimed to investigate whether naturally-acquired memory T cell responses to TRAP or CS, as measured by cultured ELISPOT, and other cell-mediated immune parameters, are associated with malaria incidence in the field.

Stratification of responses by age was carried out to determine whether immune response, as resistance to infection or disease, is simply acquired over time. TRAP cultured responses were established early, during the first 5 years of life, and did not significantly increase thereafter. In contrast, responses to CS developed more slowly with a significant rise over time. The *ex-vivo* responses showed a similar pattern of development of response over time, although the number of individuals examined >20 years were few. These data suggest that TRAP may be a more immunogenic antigen for T cell responses than CS since responses to it are generated more rapidly. Indeed, this is consistent with the weaker immunogenicity observed for CS compared to TRAP when DNA and viral vector vaccines were used in prime-boost regimens both in the UK [24], [25] and in the field [26], [27].

A comparison of antigen-specific responses showed that *ex-vivo* TRAP and CS responses correlated significantly, suggesting recent simultaneous exposure to these antigens in responding individuals. However, *ex-vivo* responses did not correlate with cultured responses to the same antigen, either for TRAP or CS, confirming our earlier findings [18]. There was a trend for cultured TRAP and CS responses to correlate, perhaps reflecting their differences in intrinsic immunogenicity but suggesting simultaneous exposure. Investigation of the phenotype of responding cells by ICS revealed a predominance of CD4^+^ IFNγ^+^ cells against TRAP, again similar to our previous findings [12], [23], compared to a more balanced CD4^+^/CD8^+^ composition for responses to CS.

In order to examine the relationship between immune parameters and malaria incidence, analysis models used survival as a continuous variable or logistically, and included age and all responses as co-variates in multivariate analysis (Table 2). Age is particularly important as resistance (“protective immunity”) to disease and infection develops over time, potentially mediated by numerous factors in addition to cell-mediated immunity. Throughout all the analyses the phenotypes consistently associated with subsequent malaria incidence were cultured TRAP response, and the CD4^+^CD25^high^ population, but there was also weaker evidence of some association with the CD56^dim^ population and reduced incidence. The cultured TRAP response was significantly associated with reduced malaria incidence. Depletion studies have demonstrated that the responding cells in cultured IFNγ ELISPOT possess the central memory T cell marker CCR7 prior to culture [17], [S Todryk unpublished]. Therefore, this suggests that the possession of such memory T cells against TRAP provides a protective advantage against malaria which would be generated by multiple contacts with antigen and remain at detectable levels once antigen levels have dropped, while the effector T cell numbers decline. These memory T cells become activated to proliferate and differentiate into effector cells upon re-exposure to antigen during a subsequent liver stage infection. Thus, a population of IFNγ-secreting T cells are provided that are capable of interfering with the liver stage of the life cycle, thus reducing or preventing merozoite release. The fact that the relationship between these cultured TRAP responses and malaria incidence is only moderately significant may not be surprising as many other factors are likely to contribute to protection in the field. These could include T cells reactive to other liver-stage antigens (although not CS, as shown in this study), antibody responses and innate mechanisms, and immunity to other stages of the life-cycle. Interestingly, the level of TRAP cultured ELISPOT response associated with protection here is in the range currently achievable by vectored vaccination regimes, at least in UK vaccinees, of around 400 SFC/M [19]. There was a difference between these findings and our recent studies (in conjunction with a vaccine trial [26]) in a neighbouring region of Kenya, where responses in non-vaccinated children were lower and not associated with protection. This may be due to reduced overall incidence in malaria in 2005 compared to 1998, as well as the locational difference, giving rise to less less T cell priming and not posing as testing a challenge as in 2005.

We found some weak evidence that increased CD56^dim^ populations may be associated with reduced malaria incidence. CD56^dim^ NK cells are known to be cytolytic [1] and so may have activity against host cells harbouring parasites and expressing parasite antigens or stress molecules.

Lastly, individuals with higher numbers of CD4^+^CD25^high^ T cells were significantly associated with increased susceptibility to malaria. Cells of this phenotype will vary randomly within any human population dependant on a variety of extrinsic and intrinsic factors. The T cells are usually considered regulatory T cells when they express high levels of FOXP3. Such cells are known to inhibit proliferation of T cells in an antigen non-specific manner and may therefore inhibit parasite-specific T cells, as has been demonstrated in mouse [4] and human [5] malaria challenge models. In view of the many potential immune responses that could be affected by this CD4^+^CD25^high^ cell population, further detailed analysis of this population in natural malarial immunity is warranted. We found no negative correlation between levels of these cells and levels of cultured ELISPOT reactivity against TRAP or CS suggesting an absence of inhibitory effects on these T cell responses, at least *in vitro*. The CD25^high^ T cells could be affecting other T cell specificities, or other arms of the immune response.

Overall, these findings support a role of cell-mediated immunity in resistance to malaria infection, in particular T cell memory against TRAP. They also highlight the probability that both multiple adaptive and innate mechanisms together contribute to an individual's susceptibility to infection and/or disease. This complex relationship warrants further investigation in larger studies if we are to further dissect the mechanisms of natural protective immunity.

## Supporting Information

Figure S1 Dot plots of individual responses against TRAP or CS measured by ex-vivo and cultured ELISPOT
(0.20 MB PPT)

Figure S2 Controls used in FACS analysis
(0.04 MB PPT)

Text S1 Ethics Approval
(0.25 MB PDF)

Text S2 Consent Form
(0.03 MB DOC)
