# Correction: Correlation of Memory T Cell Responses against TRAP with Protection from Clinical Malaria, and CD4^+^ CD25^high^ T Cells with Susceptibility in Kenyans

**DOI:** 10.1371/annotation/23d1eb6a-45de-4181-a6dd-eff9b08d2669

**Published:** 2011-02-26

**Authors:** Stephen M. Todryk, Philip Bejon, Tabitha Mwangi, Magdalena Plebanski, Britta Urban, Kevin Marsh, Adrian V. S. Hill, Katie L. Flanagan

The following information was missing from the Funding section: "The study was supported by funding from the NIHR Oxford Biomedical Research Centre programme."

