# Supplementary material for: Correlation of Memory T Cell Responses against TRAP with Protection from Clinical Malaria, and CD4+ CD25high T Cells with Susceptibility in Kenyans
Source: PLoS One. 2008 Apr 30;3(4):e2027. doi: 10.1371/journal.pone.0002027 (PMC2323567; doi:10.1371/journal.pone.0002027)
Supplement: Figure S1 — Dot plots of individual responses against TRAP or CS measured by ex-vivo and cultured ELISPOT (0.20 MB PPT) [file pone.0002027.s001.ppt]

## Slide 1
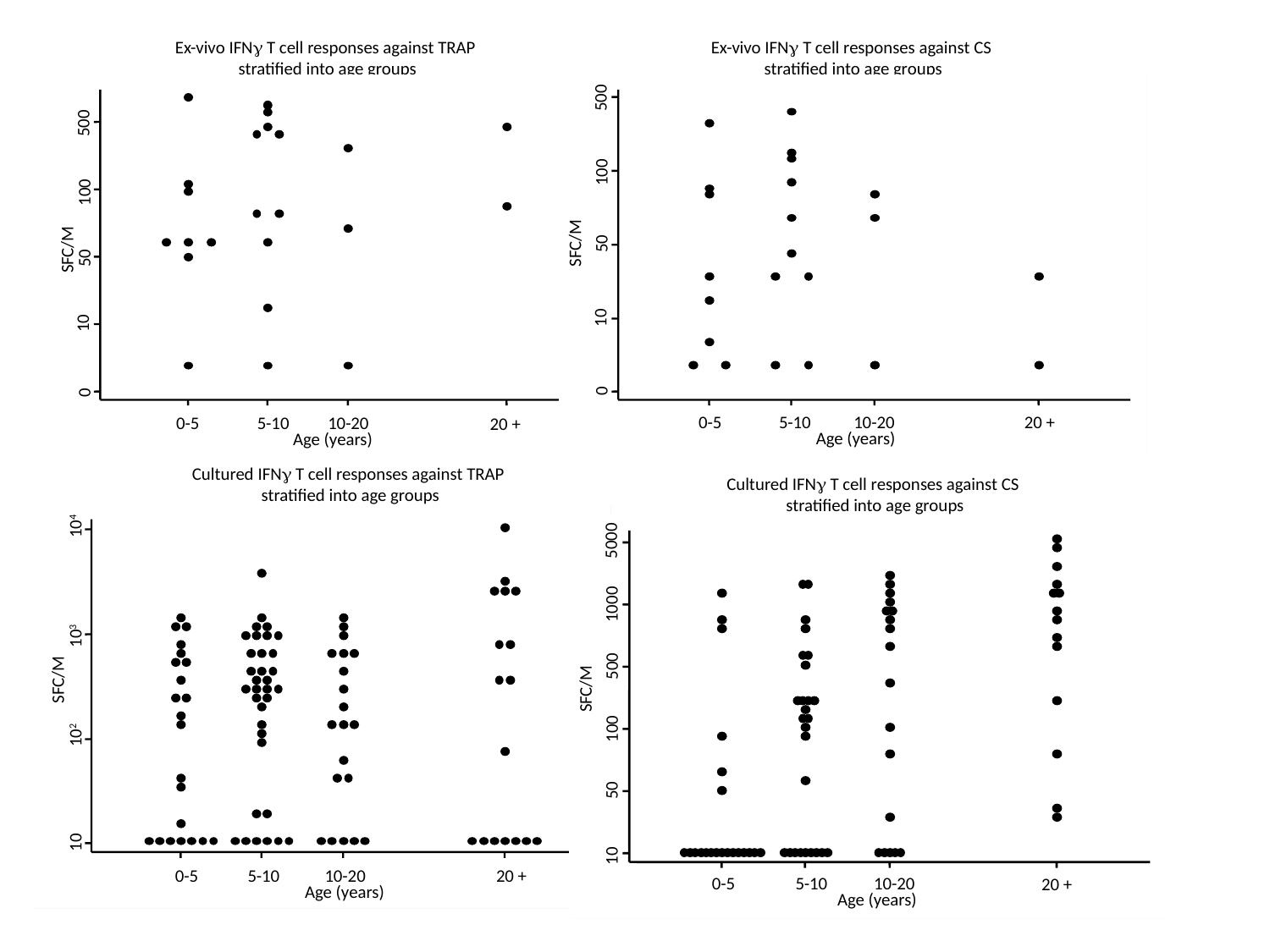

Ex-vivo IFNγ T cell responses against TRAP
stratified into age groups
Ex-vivo IFNγ T cell responses against CS
stratified into age groups
500
500
100
100
SFC/M
50
SFC/M
50
10
10
0
0
0-5
5-10
10-20
20 +
0-5
5-10
10-20
20 +
Age (years)
Age (years)
Cultured IFNγ T cell responses against TRAP
stratified into age groups
Cultured IFNγ T cell responses against CS
stratified into age groups
104
5000
1000
103
500
SFC/M
SFC/M
100
102
50
10
10
0-5
5-10
10-20
20 +
0-5
5-10
10-20
20 +
Age (years)
Age (years)
