# Supplementary figures and images for: Correlation of Memory T Cell Responses against TRAP with Protection from Clinical Malaria, and CD4+ CD25high T Cells with Susceptibility in Kenyans
Source: PLoS One. 2008 Apr 30;3(4):e2027. doi: 10.1371/journal.pone.0002027 (PMC2323567; doi:10.1371/journal.pone.0002027)

## Slide 1
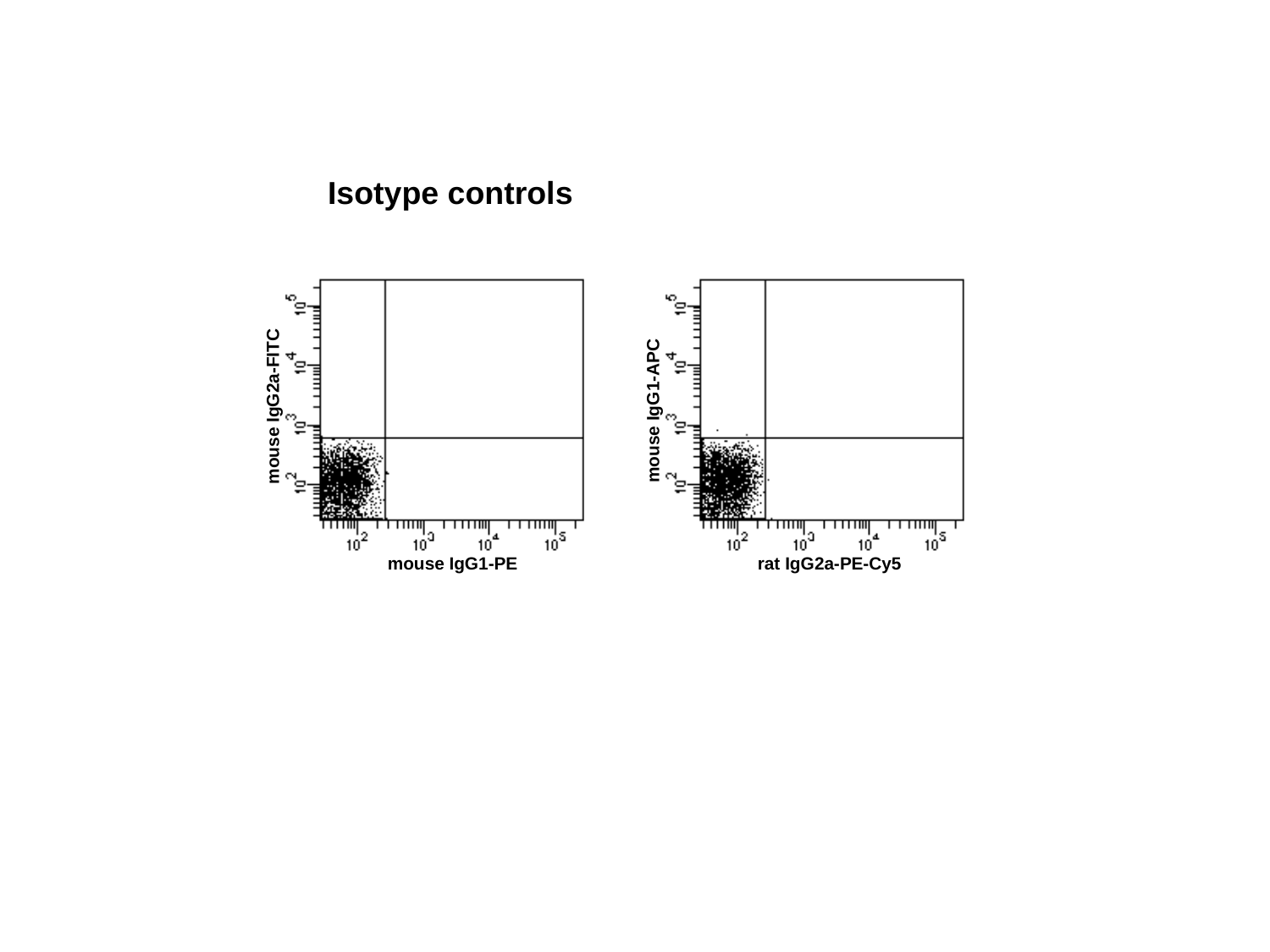

Isotype controls
mouse IgG2a-FITC
mouse IgG1-APC
mouse IgG1-PE
rat IgG2a-PE-Cy5

Supplement: Figure S2 — Controls used in FACS analysis (0.04 MB PPT) [file pone.0002027.s002.ppt]
