# Supplementary material for: Correlation of Memory T Cell Responses against TRAP with Protection from Clinical Malaria, and CD4+ CD25high T Cells with Susceptibility in Kenyans
Source: PLoS One. 2008 Apr 30;3(4):e2027. doi: 10.1371/journal.pone.0002027 (PMC2323567; doi:10.1371/journal.pone.0002027)
Supplement: Text S1 — Ethics Approval (0.25 MB PDF) [file pone.0002027.s003.pdf]

KENYA MEDICAL RESEARCH INSTITUTE

P.O. Box 54840, Tel: (02) 722541, Fax: (02) 720030, Tlx. 25696 KEMRI, NAIROBI, Kenya.

KEMRI/RES/7/3/1

9th June, 1995

Dr. K. Marsh,
CRC - Kilifi Unit

Thro'

The Director,
Clinical Research Centre,
NAIROBI

Forwarded
NCfw 12/6/95

RE: SSC Protocol No. 359 "The role of Plasmodium falciparum variant antigens expressed at the infected cell surface in generation of protective immunity and in the pathogenesis of clinical malaria" by K. Marsh et al.

During the 64th KEMRI/National Ethical Review Committee meeting held on 6th June 1995 in the KEMRI Headquarters Lecture Room the above protocol was granted full approval since you presented an acceptable Informed Consent Form.

G.A.O. ISEKO
FOR: SECRETARY,
KEMRI/NATIONAL ETHICAL REVIEW COMMITTEE
