# Supplementary material for: Correlation of Memory T Cell Responses against TRAP with Protection from Clinical Malaria, and CD4+ CD25high T Cells with Susceptibility in Kenyans
Source: PLoS One. 2008 Apr 30;3(4):e2027. doi: 10.1371/journal.pone.0002027 (PMC2323567; doi:10.1371/journal.pone.0002027)
Supplement: Text S2 — Consent Form (0.03 MB DOC) [file pone.0002027.s004.doc]

# APPENDIX IV

**INFORMED CONSENT**

**Title of Study: The Natural History Of Acquired Immunity To Malaria.**

**Investigators:** Prof. Kevin Marsh Dr. Tabitha Mwangi, Samson Kinyanjui, Victor Odera, Dr. Peter Bull, Dr. Bob Snow,

We would like to do a study to try and improve the diagnosis, treatment and prevention of malaria. In this study we are interested in trying to understand two things about malaria. Firstly we want to know the various symptoms that accompany the mild malaria fevers that are treated in outpatient clinics or at home without admission to hospital. Secondly would like to study the way the body’s ability to resist infection by malaria parasites develops as a person becomes older.

To do this we need to follow up a group of children and adults for a year during which period we will record all cases of fever and the presence of malaria parasite in their blood. We are requesting you to participate in this study.

At the start of the study you will be provided with bus fare to come to Kilifi District Hospital where you shall be examined for fever and also asked to give a small volume of blood to be examined for malaria parasites . After this you will be followed up for a year. During this period KEMRI workers will visit you once a week and take your body temperature. If you have fever , a small blood sample will drawn from you by finger prick to make a malaria slide. You will be given busfare to go to Kilifi District Hospital where you will receive treatment and also busfare for your journey back home. You will also be requested to give small volume of blood two weeks after treatment. All volumes of blood will be taken by a trained personnel. A special cream be applied on the part to be pricked to reduce pain.

A blood smear will also be made if you have had fever at any time since the last visit by KEMRI workers even though you are well during the current visit, If malaria parasites are found on the smear, the workers will bring you antimalarial drugs the next day.

You/your child will benefit in having continuous examination for fever and malaria parasites and receiving prompt treatment for any disease detected. The results of this study will help us in understanding how we can best diagnose, treat and prevent malaria. However you participation is purely voluntary and you will in no way be penalized for refusing to participate. You will also be free be withdraw from the study anytime you wish without any explanation. Please feel free to ask any questions about the study and if after this discussion you have decided to participate in this study, we would like you to sign the accompanying form

I, __________________________ confirm that _________________________ has explained this study to me in Kiswahili / Kigiriama /English which is a language in which I am fluent. In giving consent to this study I understand that refusal or withdrawal of consent will in no way prejudice my treatment, and that I may withdraw from the study at any time.

Signature or thumb print _____________________ Date ____________________

Name of person obtaining consent ____________________ Signature______________________

###### Investigator__________________
